# Innovation in medical education: a culinary coaching tele-nutrition training program

**DOI:** 10.1080/10872981.2018.1510704

**Published:** 2018-08-29

**Authors:** Rani Polak, David Pober, Adi Finkelstein, Maggi A. Budd, Margaret Moore, Julie K Silver, Edward M Phillips

**Affiliations:** a Department of Physical Medicine and Rehabilitation, Harvard Medical School, Joslin Diabetes Center and Institute of Lifestyle Medicine, Spaulding Rehabilitation Hospital, Boston, MA, USA; b Lifestyle Medicine Center, Sheba Medical Center, Tel Hashomer, Israel; c Department of Medicine, Harvard Medical School, Joslin Diabetes Center, Boston, MA, USA; d Department of Nursing, Faculty of Life and Health Sciences, Lev Academic Center, and Program of Medical Humanities, Hebrew University Hadassah-Medical School, Jerusalem, Israel; e VA Boston Healthcare System, Spinal Cord Injury Service, Boston, MA, USA; f Institute of Coaching, McLean Hospital, Harvard University Extension School, Wellesley, MA, USA; g Department of Physical Medicine and Rehabilitation, Harvard Medical School, Spaulding Rehabilitation Hospital, Boston, MA, USA; h Department of Physical Medicine and Rehabilitation, Harvard Medical School, Massachusetts General Hospital; and Brigham and Women’s, Boston, MA, USA; i VA Boston Healthcare System, Physical Medicine & Rehabilitation Service, Boston, MA, USA

**Keywords:** Medical education, nutrition, home cooking, health coaching, culinary coaching

## Abstract

**Background**: Nutrition medical education training programs that are focused on home cooking are emerging. **Objective**: This short communication describes the first synchronous tele-nutrition medical education training program using a novel Culinary Coaching (CC) model. **Design**: Seven health coaches were trained and each coach delivered CC programs to four patients (28 total). Evaluations included:1) two questionnaires before, immediately after, and six months post training program; and 2) one questionnaire after each patient program. **Results**: CC training significantly improved coaches’ attitudes about and confidence to deliver CC from pre-program means of 3.61 and 3.65 (out of 5), respectively, to post-program means, 3.77 (p<0.01) and 3.86 (p<0.05), respectively, and remained higher 6 months after the training program (3.93, p<0.01; 3.93, p<0.05). Health coaches described a high usage of CC principles and tools through the patient programs. **Conclusions**: This early evidence suggests that the CC model can be successfully expanded to health coaches, thus improving nutritional care.

## Introduction

Nutrition medical education training programs that focus on home cooking as a way to improve nutrition are emerging [1]. This is important given that an unhealthy diet is associated with 26% of annual deaths in the US [2]. Experts recommend improving nutrition education by combining a focus on nutrients along with skill-based education, such as shopping and meal preparation [3]. However, a recent review identified gaps in existing programs [1]: (1) curricula center on culinary skills rather than common barriers to home cooking, such as low confidence in cooking skills, and inadequate time and management skills [4]; (2) a majority of curricula lack an adequate behavioral change component that addresses the challenges of initiating and sustaining home cooking habits; and (3) most programs include hands-on modules in teaching kitchens, which may be ideal, but present barriers for scalability including low accessibility and high costs. In response, our goal is to develop a low-cost, attainable nutrition medical education program that efficiently and effectively addresses common home-cooking barriers through culinary skills training combined with behavioral change techniques.

The culinary coaching (CC) was developed to improve participants’ nutrition through a two-pronged approach of combining culinary training and health coaching [5]. Health and wellness coaching has proven to be effective in promoting nutritional change [6] and has developed national standards and accreditations [7]. Culinary Coaching is defined as *‘a behavioral intervention that aims to improve nutrition and overall health by facilitating participant’s home cooking through an active learning process that combines culinary training with health and wellness coaching competencies’* [8]. This report describes the feasibility and potential use of the first synchronous remote medical education training program using CC to improve nutrition.

## Methods

The CC training program was developed as a remote synchronous medical education training program aimed at educating health coaches to improve patients’ home cooking habits. Competencies were set within each category of the health coaching competencies (i.e., coaching structure, coaching process, health and wellness, ethical and legal) with a focus on trainees’ personal behavior as a lever for professional activities [9]. The synchronous remote training program included 8 weekly live sessions delivered using Google Hangout, combined with asynchronous open access culinary resources, curated specifically for the program (e.g., recipes and videos) [10]. Each weekly session included a 60-min lecture on specific culinary skills and coaching principles; and a 30-min practice session of culinary goal setting (including using the culinary resources), goal review, and culinary brainstorming. Discussions were facilitated by both the program faculty (a physician who is also a chef and health coach) and health coach trainees (supported by faculty feedback) and included reflections about how the trainees’ experiences can improve their competencies. Culinary content areas included healthier ingredients and culinary skills, home cooking during the day, food purchasing, and kitchen workflow. Health coaching content areas included advanced coaching principles which are focused on home cooking including setting culinary goals using SMART (Specific, Measurable, Achievable, Realistic, Time-based), culinary training in coaching, food language, and appreciative inquiry in home cooking (Online Supplementary Appendix describes the updated remote training program in CC curriculum).

Seven (for an effective group coaching) certified health coaches with culinary credentials from seven North American sites were recruited through social media. Inclusion criteria: (1) Health and Wellness Coaching certification, (2) culinary credential (or a proven 5 years’ culinary experience), and (3) active coaching practice where patient programs could be delivered. All seven recruited coaches completed the training program. In addition, each health coach completed a 6-month internship where each implemented the CC in four programs with patients with cardio-metabolic risk factors from her practice [8,11] (including one mentored program, in which the faculty was an observer in three sessions and provided feedback). Each month the coaches and CC faculty conducted 1-h remote group sessions to discuss challenges and successes.

Program evaluation included engagement in both the training program and the patient coaching program, and a mixed method evaluation through three questionnaires:

1. Coaches’ personal culinary behaviors were measured at baseline, after the training program, and after the 6-month internship by the validated Cooking with Chef Questionnaire [12]. This included four Likert-scale items assessing negative attitude regarding cooking (1 = strongly disagree; 5 = strongly agree), and 13 Likert-scale items assessing confidence to cook (1 = not at all confident; 5 = extremely confident);
2. Coaches’ professional behaviors were measured at baseline, after the training program and after the 6-month internship by the Training Questionnaire. This included five Likert-scale items assessing attitudes regarding home cooking education (1 = do not agree at all; 4 = highly agree), seven Likert-scale items assessing confidence to deliver home cooking education using coaching principles (1 = not at all confident; 4 = very confident), and open-ended questions assessing the overall training program, and motivation and expectations from patients’ programs.
3. Implementation of the training content areas was measured by a Program Questionnaire after each of the four patient programs. The questionnaire included nine Likert-scale items assessing the usage of key CC culinary principles and communication strategies that were discussed in the training program, and open-ended questions assessing the patient program challenges and the training program contribution to provider competencies.

Items for questionnaires 2 and 3 were revised from questionnaires assessing the feasibility of a web-based professional training [13], attitudes regarding home cooking education [14], and health coaching [15].

Quantitative responses were tested across time points by linear mixed effects model (analogous to repeated measures ANOVA). Qualitative descriptive analysis [16] was conducted by a qualitative researcher and the program’s Principal Investigator. We searched for the main elements of learning in the participant experience. We looked for similarities and differences in answers within and across the seven participant coaches. We conducted several iterative cycles of coding and discussing emerging themes and adjusting codes until we reached agreement on the final categories. There were no disputes requiring a third party. This report was determined as not human subjects’ research by the Joslin Diabetes Center Committee on Human Studies and exempt from further review.

## Results

Health coaches improved negative attitudes about and self-efficacy to perform various home cooking activities from a mean (standard deviation) of 1.22 (0.51) (out of 5, lower is more positive) and 4.64 (0.62) (out of 5, higher is more positive), respectively, before the training program to 1.07 (0.46) (*p* < 0.05) and 4.88 (0.69) (*p* < 0.01) after the training program, and remained improved 6 months after the training program, 1.11 (0.49) (*p* < 0.05) and 4.86 (0.74) (*p* < 0.01). Further, coaches’ attitude about and confidence to use the CC model improved from pre-program means of 3.61 (0.68) and 3.65 (0.57) out of 5 (higher is more confident), respectively, to post-program scores of 3.77 (0.63) (*p* < 0.01) and 3.86 (0.87) (*p* < 0.05). Scores remained higher 6 months after the training program (3.93 (0.56) (*p* < 0.01) and 3.93 (0.81) (*p* < 0.05)). These results are reflective of, for example, 4/7 participants responding that they were very confident about home cooking activities before training program compared to 6/7 after training program (Cohen’s effect sizes ~0.9–1.1).

Twenty-two (79% of the 28) patient programs were completed during the program’s internship. Table 1 describes the extent to which providers implemented key CC principles in the 28 patient programs. In 23 patient programs, providers reported 75–100% completion of culinary goals, and in four patient programs, 50–75%. In 20 programs (71%), coaches reported that patients obtained additional nutritional information during the program through their provider (e.g., physician, dietitian).

**Table 1.** Use of culinary coaching content areas and strategies during the patient programs.

	1	2	3	4
**How often did you discuss the following culinary content areas with your patient?**				
Healthier ingredients and culinary skills (mean (SD) = 3.64 (0.62))	0%	7%	21%	71%
Home cooked food during the day (3.64 (0.49))	0%	0%	36%	64%
Food purchasing (3.36 (0.78))	0%	18%	29%	54%
Efficient kitchen workflow (3.50 (0.75))	0%	14%	21%	64%
Healthier cooking strategies (3.29 (0.66))	0%	11%	50%	39%
**How often did you use the following health coaching strategies with your patient?**				
SMART culinary goals setting (3.69 (0.61))	0%	7%	18%	75%
Culinary training using coaching principles (3.43 (0.69))	0%	11%	36%	54%
Food language (food items vs. nutrients) (3.32 (0.77))	0%	18%	32%	50%
Appreciative inquiries (3.82 (0.55))	0%	7%	4%	89%

Percent of responders’ perceived usage of culinary content areas/health coaching strategies throughout the culinary coaching programs, *n* = 28 (1 – none of the sessions; 4 – all the sessions). Mean and standard deviation (SD) are presented for each question as well.

Coaches’ perceptions from the training program were positive with notable themes: (1) The CC’s two-pronged approach was praised as a powerful tool. However, graduates perceived navigating between training and coaching as challenging. *‘The shift from “coach” to “trainer” is the area I want to strengthen. Ideally the transition would be seamless*.’ (2) The practice sessions were one of the training program highlights. Participants praised their learning experience from both practicing and observing the program faculty as well as their colleagues. ‘*I am now even more aware of my strengths and weaknesses, my skills as a chef and a coach and how that relates to the manner in which I present guidance to clients, and how I see myself as an agent of change.’* (*3)* Participants reported a beneficial educational experience from using culinary videos for their own education as well as a potential resource for patients. *‘These videos could also be a resource for clients.’*

## Discussion

This pilot demonstrates that a synchronous remote medical education training program in CC is feasible, and was well-received by trainees who reported improved personal culinary behavior and confidence to use CC. These results are consistent with reports of onsite culinary trainings which are shown to improve providers’ personal and professional outcomes [17]. The value of medical education training program in CC was further supported by (1) coaches continuing to use the CC model after the internship completion; (2) Spaulding Rehabilitation Hospital adopting the training program as a professional training for clinicians; (3) Wellcoaches School of Coaching’s approval of the training program for continuing education credits; (4) Harvard Medical School’s approval of the training program for Continuing Medical Education credits; and (5) life insurance authorization of patient programs for patient with long-term disability.

The curricular principles can also be adapted to address a variety of educational needs. For example, The American College of Preventive Medicine developed a short 2-h remote asynchronous training program in CC for their national training [18]. We are also currently adapting this model to address the needs of both the geriatric population and women with breast cancer.

The CC tele-nutrition training program has potential limitations that might impact viability such as individuals without access and/or skills to use internet-enabled devices. Also, further research is required to evaluate the impact of CC on culinary behaviors, health outcomes and healthcare costs of patients with various health conditions. This study used a small sample of convenience and larger studies are needed for confirmation. Our goal is to determine whether the remote CC initiative can make an efficient, effective, and scalable impact in supporting clinicians and health coaches in delivering home cooking education, and therefore, providing value-based preventive care.

## Data Availability

This report data is available through the corresponded author.

## Supplementary data

Supplementary data can be assessed here.
