# Supplementary material for: Innovation in medical education: a culinary coaching tele-nutrition training program
Source: Med Educ Online. 2018 Aug 29;23(1):1510704. doi: 10.1080/10872981.2018.1510704 (PMC6127849; doi:10.1080/10872981.2018.1510704)
Supplement: Supplemental Material [file ZMEO_A_1510704_SM0471.docx]

Appendix 1: Culinary coaching tele-nutrition training program

Learning objectives

Participants will be able to:

- Define the relationship between home cooking and health and discuss the rationale for the importance of patients’ culinary behaviors.
- Demonstrate strategies for facilitating patient-centered culinary knowledge and skills, and describe strategies for empowering patients to adopt home cooking.
- Develop their own culinary confidence and skills, and learn strategies to facilitate cooking for improved personal health.
- Use culinary resources to improve culinary behaviors of physicians/clinicians and their patients.
- Employ culinary coaching telemedicine programs with their patients.

Syllabus

**Skill development sessions**

| **Introduction to Culinary Medicine:** The Health and Social Benefits of Home Cooking: What the Newest Data Clearly Shows Us |
| --- |
| **Introduction to Lifestyle Medicine**: Incorporating Culinary Medicine as Part of a Comprehensive Lifestyle Approach |
| **Healthier Ingredients and Culinary Skills *:** What and How Should My Patient Cook?* |
| **Why Doesn’t My Patient Cook?** Recognizing and Making It Easier for Patients to Address the Reasons They Don’t Embrace Healthier Cooking Practices |
| **Mitigating Cardio-Metabolic Risk Factors*:** Checklists for Shopping, Pantries, Kitchen Tools, and Utilizing Ingredients* |
| **Getting Patients (Even Reluctant or Unskilled Cooks) Into the Kitchen:** Motivational Interviewing and Goals Setting to Produce Healthier Kitchen Behaviors |
| **Cooking with Little Time or Budget*:** Kitchen Economies and Efficiencies to Make Healthier Kitchens More Accessible to More Patients |
| **Mastering Home Cooking*:** Advanced Healthy Cooking Strategies to Improve Home Cooking for You and Your Patients |
| **In-Office Culinary Education Tools and Delivery Strategies**: Group Lectures, Individual Coaching, Hands-On Instruction, and Reimbursement Strategies |
| **Mastering Culinary Coaching**: How to Quickly and Effectively Help Patients Set and Achieve Realistic Culinary Goals |

* Skill development sessions which also include recipes, introduction to new cooking techniques, and several tips to improve your home cooking.

**Small group practice sessions**

| **Putting Everything Together: Recipe for Success:** Improve your own home cooking and your culinary coaching skills in small groups led by program faculty. Explore new culinary practices in your own kitchen, practice culinary coaching strategies with your colleagues, and improve your competencies to prescribe nutrition through reflections with the group facilitator. |
| --- |
